# Salivary Calculi

**Published:** 1898-03

**Authors:** W. Freudenthal

**Affiliations:** President of the German Medical Society of the City of New York, New York, N. Y


### Salivary Calculi.

Presented to the Section on Laryngology and Otology, at the Forty-eighth Annnal Meeting of the American Medical Association, held at Philadelphia, Pa., June 1-4, 1897.

BY W. FREUDENTHAL, M. D.,
President of the German Medical Society of the City of New York, New York, N. Y.

Salivary calculi are, as a rule, easily recognized, although they are not of frequent occurrence. They are most frequently located near the buccal end of Wharton's or Steno's duct, and often have a definite outline and shape, rendering their detection by palpation usually easy. There are, however, a number of cases recorded in which their discovery was made by mere chance. Such an instance occurred in my practice. In two of my cases the diagnosis was easily made.

It appears that the growth and formation of salivary calculi begin years before their detection, and in this respect their character is quite in contrast to the stones found imbedded in the nasal cavities, which latter reach a large size after sometimes a growth of only a few months. The secretory function of Steno's or Wharton's duct and of the nasal cavity proper are not only different, but the foreign bodies found there are of different sizes. Sometimes these ducts contain large stones, without in any way producing symptoms, or in any sense annoying the patient. Such an instance is the following case:

*Case* 1.—Mr. A. F., 23 years of age, for the last six years a professional clarionetist, came to me last winter, in the height of his season, complaining of a cold, contracted two weeks before, and in consequence of this an abscess had formed, as he believed, under the tongue. On examination I found a swelling about the size of a hen's egg in the left sublingual region. No hard substance could be felt at all. Believing that I was dealing with a ranula, I made a long incision through the buccal mucosa. A mass of feted pus escaped, and, to my surprise, the end of a stone, oblong in shape, appeared in the opening. It was readily extracted. It undoubtedly came from Wharton's duct, judging from its contour. There is nothing unusual in its size and weight.

After thoroughly cleansing the abscess cavity I passed a probe and was surprised to find evidence of another stone situated deeply within the tissues of the submaxillary gland. My first effort to extract it resulted in failure, as it slipped back further in the cavity. My second attempt however was successful. The stone was irregularly oval and about as large as a cherry. One would suppose that a person whose mouth is constantly in action by reason of his professional occupation, would be able to detect anything of an abnormal character in it. Yet in this instance the patient never suspected anything whatever, until two weeks ago when as he states, he contracted the cold, which most likely caused the abscess about which he consulted me. He is positive that before contracting his cold he never felt anything unusual in his mouth.

As an illustration of the powers of endurance, it is interesting to state that my patient, a weak man at best, not only followed his usual vocation as a clarionet player up to the day on which the calculus was discovered and extracted, but also played his musical instrument on the evening after the operation despite the fact that there was a large iodoform tampon in the old site of the calculus. There are on record other instances where a supposed "cold" led to the detection of salivary calculi.

Mr. Carver * removed a stone from a man 70 years of age, who had noticed some tenderness in the sublingual region only for about five or six weeks.

Demons † found a stone the size of an olive in Wharton's duct, which produced no symptoms until three weeks' before its removal.

There are, on the other hand, cases recorded in which the stone had made its presence felt for several years before its final extraction. Alfred Smith and C. T. Scott § reported a case in which a tumor had been first noticed by the patient seven years before, at which time it was the size of a pea.

*The Lancet, March 13, 1886, p. 494.

†Jour. de Med. et de Chir. de Bordeaux, Sept. 6, 1885.

§Jour. of the British Dental Assoc., 1888, p. 175.

Jos. Dixon * describes the sufferings of his wife, who had noticed a swelling under the tongue for fifteen years. Mr. Alexander Bruce † removed a stone of "considerable dimensions," as he states it, although it weighed 11½ grains. In this instance the patient had felt it fourteen years previously. Dr. Webb's ‡ patient felt a small lump sixteen years before being operated upon. Dr. Padieu's § patient felt it for twenty-five years, and Dr. Shuster's || patient for forty-four years (!)

It is known that patients suffering from salivary calculi frequently obtain relief from their symptoms by squeezing out the pus and mucus which surround the stone and fill the cavity, into the mouth, from which they expectorate it or unconsciously swallow it. Thus, we can understand in Jos. Dixon's case the attacks of dyspepsia lasting for fifteen years, which were not amenable to treatment. The patient apparently swallowed the secretions which she pressed out from the glands.

Other symptoms caused by the stones are pain increased by movements of the jaw such as during mastication, and from retention of saliva shortly after mastication. Still another sympton occasionally encountered is difficulty in breathing. That this last symptom is sometimes dangerous to life is proven by the case of J. W. Hulke, and my second case.

Hulke ¶ treated a young lady who had a tumor under the tongue which had been growing for the last six years. This tumor sometimes enlarged temporarily to such proportions as to threaten suffocation. At times there would be a discharge into the mouth, followed by the return of the swelling to its former size. The patient indicated by her statements that the stone had nearly suffocated her.

The following case which came under my observation, illustrates this last point:

*Case* 2.—Mr. N. S., aged 45 years, had been suffering two and a half years before he noticed a swelling under the tongue.

* Lancet, Nov. 26, 1887, p. 1063.
† Transactions of the Pathol. Soc. of London, Vol. xvii. p. 134.
‡ Ibidem, viii. p. 221.
§ Gazette Med. June 6, 1885.
|| Oester. Vierteljahrsschr. fuer Zahnheilkunde, Oct. 1886.
¶ Transactions of the Pathol. Soc. of London, Vol. xxiv. p. 88.

This swelling grew steadily, and occasionally caused him great pain. For the last ten days the pain and swelling had grown worse. During the night he snored so loudly that he had to be wakened, and on these occasions his face would become markedly cyanotic. The patient was tall and very stout, and had the hoarse voice that characterizes the chronic alcoholic. On examination the tumor was found to project slightly from under the tongue and to extend along the right of the frenulum, reaching to the floor of the mouth, backward between the root of the tongue and the angle of the jaw, at which spot it appeared to be adherent to the buccal mucous membrane, simulating the case described by Hulke. By palpation fluctuation was elicited, and a hard mass could be plainly felt within it. I was quite sure that we had to deal here with a salivary calculus which had formed in the beginning of the main duct, and I advised its removal. For obvious reasons the patient refused and went home. His heart and lungs were normal.

Several weeks later I received a note from his physician, stating that the patient had died. The night preceding his death he had imbibed more freely than usual, and then retired. Having fallen asleep, he began to snore so loudly that his wife awoke. She found the patient breathing with great difficulty, while his face was black and blue. She tried to awaken him, but failed. She had a physician called ; but the patient died of suffocation before medical aid reached him. An autopsy disclosed an almond-shaped salivary calculus, imbedded in a pus cavity. Death no doubt resulted indirectly from its presence; for on previous occasions he would invariably get rid of the pus that had accumulated in the cavity by coughing or hawking it out, and in this manner relieve himself of the cause of his dyspnea. But on the fatal night he was so fully under the influence of liquor that he could not arouse himself from the stupor caused by it, and thus succumbed.

I remarked in the beginning of this paper, that when I compared the sizes of salivary calculi and rhinoliths that I believed that the majority of these deposits owe their formation to the fact that a foreign body enters the duct or gland itself.

When Clinton Wagner * says: "These concretions are formed by the deposit of earthy salts from the saliva in the excretory ducts leading from the gland or in the body of the gland itself, and that the cause of the deposit is an obstruction to the flow of saliva either to or through the excretory duct," he overlooks the primary cause of their formation *i. e.*, a foreign body. Around this foreign body is deposited layer after layer of earthy salts. This obstruction causes a retention of the salivary secretion. I do not, however, claim that this process obtains in all instances of salivary calculi, but in the majority.

Professor Ribbert, of Bern, found that a hair of a toothbrush had forced itself into Wharton's duct, thus giving rise to the formation of a salivary calculus around it.

In several other instances the same causative element was found. Hulke removed a stone weighing 76 grains. Its broken surfaces exhibited concentric rings around a small, black, central speck. This speck was a fragment of wood.

It must be true that in certain calculi no central foreign body can be demonstrated, because it has undergone chemical changes. Galippe reported a case of spontaneous discharge of a stone from Wharton's duct. This calculus had concentric strata without a kernel being found. But he found microbes, already described by Malassez and Vignal, in these deposits.

One case is reported by Wertheimer † in which an abcess formed during eating. The patient while eating blueberries suddenly noticed a tumor in the right submaxillary region, and at the same time a burning sensation in the mouth. These symptoms always appeared during mastication. Finally, among greenish cheesy masses, the berries were found. Here stones had not yet formed, so, properly speaking, this case does not belong here, but it shows how often foreign bodies enter Wharton's duct.

*Case* 3.—Mr. M. P., 57 years of age, merchant, of moderate habits and enjoying good health, had noticed for some time a small lump in the left submaxillary region. For the last three months it had grown steadily and had become painful during and after mastication. A simple incision was made, and a small

* N. Y. Med. Jour., Nov. 11, 1893, p. 56[illegible].

† Corps Etrang. du Canal de Wharton. Bulletin Med. du Nord. Dec. 12, 1885

oval-shaped stone removed. In cutting it open, concentric rings were seen and a very small piece of wood in the center. It was ascertained that the patient had been for years in the habit of chewing toothpicks, thus most likely forcing the foreign body into Wharton's duct.

These cases certainly prove that the formation of calculi around a foreign body does occur in these as well as in other parts of the human anatomy. That there are other causes for their formation there is no doubt, and an increase or decrease in the quantity of saliva is an important factor.

A case of Thomas Ballard * is of interest: A young lady suffered from the effects of a buccal abscess which had opened spontaneously. Two small calculi were removed from the abscess cavity. It was learned that eleven years before she was salivated during an attack of jaundice. It is probable that this salivation caused a swelling of the excretory duct of the gland, then a retention of saliva and gradually the formation of a stone.

Just as increased flow of saliva may tend to produce a calculus, it may also tend to wash away a nucleus in the duct, around which salty deposits might form. This was the case with a patient of Kurtz's, who had taken pilocarpen internally. The increased glandular excretion washed out a calculus from Wharton's duct.

In conclusion, I also believe that the submaxillary is more often the site and seat of calculi than the parotid gland, because the secretion of the former gland contains a comparatively larger proportion of mucin, a substance which seems to possess considerable adhesive properties, and seems to favor the deposit of inorganic salts. This belief is confirmed by the fact that the usual site of the calculus is just at the outlet of the salivary duct on the buccal mucosa, a point which furnishes a ready ingress for foreign matter.

---

Surgeon-General Sternberg, of the United States Army, has been elected President of the American Medical Association for the coming year.

---

* Transactions of the Pathol. Soc. of London, Vol. xviii. p. 93.

